# Integrated genomic and phenotypic analysis of intra-clade variation in *Candida auris* from a New York City tertiary hospital

**DOI:** 10.1128/spectrum.01695-26

**Published:** 2026-09-24

**Authors:** Shivani Satia, Jennifer Pfeiffer, Abdullah Kilic, E. Yoko Furuya, Marie C. Smithgall, Mahesh Mansukhani, Susan Hsiao, Jun Shi, Rinki Kumar, Daniel A. Green, Gregory J. Berry, Fann Wu

**Affiliations:** 1 Department of Pathology and Cell Biology, Columbia University Irving Medical Center 21611, New York, New York, USA; 2 Department of Pathology and Genomic Medicine, Houston Methodist Hospital, Houston, Texas, USA; 3 Department of Pathology, Wake Forest University School of Medicine 12279, Winston-Salem, North Carolina, USA; 4 Department of Medicine, Columbia University Irving Medical Center 21611, New York, New York, USA; Debreceni Egyetem, Debrecen, Hungary

**Keywords:** *C. auris*, fungal pathogen, antifungal susceptibility testing, colonization, whole genome sequencing (WGS), single nucleotide polymorphism, FKS1 mutations, echinocandin resistance, *C. auris* outbreak, Intra-clade diversity, infection control, surveillance

## Abstract

*Candida (Candidozyma) auris* (*C. auris*) poses an urgent public health threat due to its widespread emergence, antimicrobial resistance, and ability to cause severe infections and outbreaks associated with high mortality rates. In this study, we phenotypically and genotypically characterized antifungal susceptibility profiles and used whole-genome sequencing (WGS) to assess overall genetic diversity and to identify clonal clusters. From July 2020 to December 2025, a total of 118 *C. auris* isolates from 97 patients were collected at a tertiary hospital in New York City. Antifungal susceptibility testing and WGS were performed on all clinical isolates to characterize their clade-specific antifungal resistance genes and intra-clade genetic variations. Ninety-nine percentage of the *C. auris* isolates demonstrated phenotypic fluconazole resistance. Resistance to echinocandins was observed in 7.6% (9/118) of isolates, and resistance to amphotericin B was found in 54% (64/118). WGS confirmed that all 118 isolates belonged to the CDC-defined Clade Ic. Azole resistance mutations in the *ERG11* and *CDR1* genes were detected in 100% (118/118) of the isolates, and mutations in the *TAC1B* gene were present in 92.4% (109/118) of the isolates, including one case with a unique set of mutations (*ERG11*-Y132F, *CDR1*-E709D, *TAC1B*-A583S). Mutations in the *FKS1* gene were identified in 5.9% (7/118) of isolates though these were not consistently linked to echinocandin resistance. Genomic analysis also revealed that individual patients frequently carried the same strain throughout the study. Furthermore, multiple strains emerged simultaneously across the hospital each year. This suggests that numerous patients were likely already colonized upon admission, rather than acquiring the pathogen through intra-hospital transmission. The presence of multiple *C. auris* strains with varying resistance patterns poses significant therapeutic challenges. Genomic typing can help hospitals understand the diversity of their *C. auris* isolates to track their spread, identify potential outbreaks, and ensure the effectiveness of infection control measures.

**IMPORTANCE:**

*Candida (Candidozyma) auris* is an emerging, multidrug-resistant fungal pathogen designated by the Centers for Disease Control and Prevention (CDC) as an urgent public threat. Traditionally, *C. auris* infections have been considered predominantly nosocomial, with outbreaks typically attributed to a single clonal lineage spreading within healthcare facilities. However, the genomic analysis performed in our study identified multiple distinct clusters of genetic variations within a single clade, suggesting that infections were not derived from a single source. This genomic diversity indicates that many patients may have acquired *C. auris* prior to hospital admission. Nevertheless, intra-hospital transmission was evident, as patient-to-patient spread facilitated the dissemination of various clonal lineages within the healthcare environment. These findings underscore the complex epidemiology of *C. auris* and highlight the urgent need for stringent infection control measures both within and beyond hospital settings.

## INTRODUCTION

*Candida auris* is a multidrug-resistant pathogen associated with hospital-acquired infections and is recognized as a significant global health threat. First identified in 2009 in the external auricular canal of a Japanese patient, it has since been detected in over 35 countries (1). It can cause a variety of infections including bloodstream, wound, surgical site, device-related, and urinary tract infections, which are more common in immunocompromised patients and associated with high mortality rates (2). Its emergence has raised serious concerns due to its resistance to multiple antifungal drugs, including polyenes, azoles, and echinocandins (3, 4).

*C. auris* primarily spreads through contact in healthcare settings. Patients can remain colonized on their skin for prolonged periods, potentially because the pathogen penetrates hair follicles and deeper skin layers and forms biofilms in the presence of sweat (5). These factors can lead to heavy shedding into the environment, causing substantial contamination of surfaces and equipment. Furthermore, *C. auris* can survive in challenging environments, making it difficult to disinfect and eliminate (6). Together, these factors facilitate further transmission to other patients in the hospital, often leading to large-scale clusters and outbreaks. In the United States, it is a nationally notifiable pathogen, and genomic surveillance has become a critical tool for monitoring and managing its spread (7).

Whole-genome sequencing (WGS) is increasingly used to identify genetic markers associated with virulence, resistance, and outbreak tracking (7). Six major genetic clades of *C. auris* have been identified, each differentiated by tens of thousands of single-nucleotide polymorphisms (SNPs). These clades are geographically distinct: clade I is associated with South Asia, clade II with East Asia, clade III with Africa, clade IV with South America, clade V with a few isolates from Iran, and clade VI recently described in Singapore and Bangladesh (8). While broad inter-clade genomic differences are well-documented, reports addressing *C. auris* intra-clade genetic and phenotypic variation remain limited (9, 10).

We previously demonstrated the use of Sanger sequencing and WGS to identify antifungal resistance in *C. auris* by targeting specific mutations in genes such as *ERG11, CDR1, TAC1B*, and *FKS1*, showing that WGS provides comprehensive tracking of entire genomes and novel resistance markers (11). We have expanded on this work by adding additional isolates collected in 2024 and 2025 to study antifungal resistance patterns using WGS and correlate them with phenotypic susceptibility results. We also utilized WGS to assess overall genetic diversity among all isolates and the dynamics of clonal clusters within the hospital over a 5-year period.

By integrating phenotypic susceptibility testing with genomic examination of resistance determinants and WGS-based SNP analysis, our study provides insights into intra-clade variation, clonal spread, and the molecular mechanisms of antifungal resistance in *C. auris*. We believe these findings will enhance awareness of emerging resistance and better inform hospital infection control strategies.

## MATERIALS AND METHODS

### Phenotypic identification and antifungal susceptibility testing

This study was conducted in a tertiary hospital in New York City from July 2020 to December 2025. A total of 118 *C. auris* isolates were collected from 97 inpatients and included both clinical and surveillance specimens. Identification was performed using matrix-assisted laser desorption/ionization time-of-flight (MALDI-TOF) mass spectrometry (Bruker Daltonics Inc., Billerica, MA) (12). Antifungal susceptibility testing was performed using Sensititre YeastOne Y09 or Y11 broth microdilution (BMD) plates (Thermo Fisher Scientific, Marietta, OH). Results were interpreted in accordance with the Clinical and Laboratory Standards Institute (CLSI) guidelines for determining susceptibility of yeasts (13) and the tentative MIC breakpoints for *C. auris* antifungal resistance established by the CDC (14).

### Whole-genome sequencing and data analysis

Whole-genome sequencing was performed on all isolates to characterize clade-specific antifungal resistance genes and intra-clade genetic variations. DNA was extracted from pure *C. auris* culture isolates per manufacturer instructions using the QIAamp DNA tissue kit (Qiagen, Valencia, CA) with a bead beating step prior to extraction.

For library preparation, 500 ng of extracted genomic DNA was fragmented to an average peak size of 400 bp by sonication (Covaris, Woburn, MA) with 10% Duty Cycle and 175 Cycles per Burst for 60 s at 4°C to 7°C. Libraries were prepared for sequencing using a PCR-free workflow (11) and the KAPA HyperPrep kit (Roche Diagnostics, Indianapolis, IN). The resulting libraries were quantified using the KAPA Universal Library Quantification Kit (Roche Diagnostics, Indianapolis, IN).

Sequencing was performed using the NextSeq2000 sequencer (Illumina, San Diego, CA) with 2 × 150 bp paired-end reads.

Genomic analysis was conducted using BugSeq’s cloud-based genomics platform (BugSeq Bioinformatics Inc., Vancouver, Canada). Fastq files were uploaded to BugSeq and analyzed using two bioinformatic pipelines. To evaluate the SNP differences between the isolates, we used a Clade I-SNP distance matrix generated by the CDC MycoSNP workflow (https://github.com/CDCgov/mycosnp). For the epidemiological analysis, a gene-by-gene approach, refMLST (15), was used to compare isolates to the *C. auris* reference genome (GenBank accession no. GCF_003013715.1). A neighbor-joining phylogenetic tree was then constructed and visualized using iTOL v7 (16).

## RESULTS

A total of 118 specimens isolated from 97 patients were analyzed, comprising 82 clinical specimens and 36 surveillance skin swabs. Of the clinical specimens, isolates were most frequently recovered from blood (*n* = 42; 35.5%), followed by urine (*n* = 21; 17.7%), wound (*n* = 8; 6.7%), respiratory tract (*n* = 7; 5.9%), and body fluids (*n* = 4; 3.3%).

### Phenotypic antifungal susceptibility

Phenotypic resistance to any echinocandin (anidulafungin, caspofungin, and micafungin) was observed in 9 of 118 isolates (7.6%). Six of nine resistant isolates were identified in urine samples. Of these, four were resistant to all three echinocandin drugs, one was resistant to anidulafungin and micafungin but susceptible to caspofungin, and two were resistant only to caspofungin. Among the other samples, one blood culture isolate and two skin swab culture isolates were caspofungin-resistant (MIC = 8 µg/mL) (Table 1).

**TABLE 1:** Phenotypic resistance of *C. auris^a^*

Source	Number of isolates	CDC tentative MIC breakpoints (µg/mL)				
		Fluconazole	Amphotericin B	Anidulafungin	Caspofungin	Micafungin
		R: ≥32	R: ≥2	R: ≥4	R: ≥2	R: ≥4
Blood	42	42	22	0	1	0
Body fluid	4	4	2	0	0	0
Respiratory	7	7	5	0	0	0
Wound	8	8	1	0	0	0
Urine	21	21	13	5	6	5
Skin swab	36	35	21	0	2	0
Total	118	117	64	5	9	5

^*a*^ Number of isolates by specimen source and antifungal resistance, based on the CDC tentative MIC breakpoints (µg/mL). MIC, minimum inhibitory concentration. R, resistant; values indicate the number of resistant isolates.

Resistance to amphotericin B was identified in 64 isolates (54.2%) (Table 1).

Among azoles, while established breakpoints for *C. auris* are lacking, the profiles indicate the following: posaconazole demonstrated the most favorable susceptibility profile, with 84.7% of isolates (100/118) exhibiting minimum inhibitory concentrations (MICs) < 1 µg/mL. In contrast, voriconazole exhibited higher MICs across most isolates (61/118, 51.7%). Additionally, 33% of isolates (39/118) and 27% of isolates (32/118) had higher MICs to isavuconazole and itraconazole, respectively. One hundred seventeen of 118 isolates were resistant to fluconazole, with phenotypic resistance levels varying from 32 µg/mL to 256 µg/mL. Only one isolate was susceptible (16 µg/mL).

### Genotypic predictions of antifungal resistance

Resistance to azoles is linked to mutations in the *ERG11*, *CDR1,* and *TAC1B* genes. As shown in Table 2, we identified a K143R mutation in the *ERG11* gene, a V704L mutation in the *CDR1* gene in 117/118 isolates, and an A640V mutation in the *TAC1B* gene in 109 (92.4%) isolates. One isolate carried a unique set of previously reported mutations (*ERG11*-Y132F, *CDR1*-E709D, *TAC1B*-A583S) (17), and a novel *TAC1B*-W387R mutation was detected in 19/118 closely related isolates.

**TABLE 2:** Genotypic characterization of *C. auris^a^*

Source	Number of isolates	Mutations associated with azole resistance (number of isolates)			Mutations associated with echinocandin resistance (number of isolates)				
		*ERG11* K143R	CDR1 V704L	*TAC1B* A640V	*FKS1* R1354H	*FKS1* R1354S	*FKS1* S639P	*FKS1* S639T	*FKS1* S639Y
Blood	42	42	42	39	0	0	0	0	0
Body fluid	4	4	4	4	0	0	0	0	0
Respiratory	7	7	7	7	0	0	0	0	0
Wound	8	8	8	7	0	0	0	1	0
Urine	21	21	21	18	0	2	2	0	1
Skin swab	36	35	35	34	1	0	0	0	0
Total	118	117	117	109	1	2	2	1	1

^*a*^ Distribution of azole and echinocandin resistance associated mutations by specimen source. The values indicate the number of isolates harboring each mutation. Note: One skin swab sample carries a unique set of azole resistance mutations (ERG11_Y132F, CDR1_E709D, TAC1B_A583S).

We also screened for additional SNPs in other antifungal resistance genes affecting ergosterol biosynthesis, transcriptional regulators, or transporter genes (File S1, sheets 1, 2) and identified the following variants: *MRR1*-K381X and *HMG1*-G765X in 3/118 isolates, *MDR1*-I341V in 18/118 and *MRR1*-KD724N in 19/118 isolates, *MRR1*-K493Ter in 10/118 isolates (File S1, sheet 1). These variants have not yet been functionally characterized. Additionally, a *FUR1*-G192R mutation in the *FUR1* gene, which drives high-level resistance to the antifungal drug flucytosine (5FC), was detected in 12/118 isolates. The isolates carrying these mutations were clustered together in a specific clone and appeared to be related.

No additional variants associated with amphotericin B resistance were identified in the *ERG* genes, including *ERG2, ERG3, ERG4, ERG5,* and *ERG6*.

The *FKS1* gene mutations associated with echinocandin resistance were present in 7 (5.9%) isolates: five from urine specimens, one from a wound, and one from a surveillance swab. Table 3 details these mutations with the echinocandin susceptibility testing results. Among the isolates phenotypically resistant to all three echinocandin drugs, four urine samples carried *FKS1* mutations (R1354S and S639P). Conversely, one wound isolate with an S639T mutation and one skin isolate with an R1354H mutation did not exhibit phenotypic resistance. One sample carrying an S639Y mutation was resistant to anidulafungin and micafungin but susceptible to caspofungin. When MIC distributions were stratified by gene mutations, a notable increase in micafungin MICs (mode: 1.0 µg/mL) was observed in nine isolates containing the *FKS1* M690I mutation, compared to the modal MIC (0.12 µg/mL) of all other isolates without the mutation (File S1, sheet 1). Furthermore, four of these nine isolates also showed phenotypic resistance to caspofungin (8 µg/mL). An additional *FKS1*-V1818I mutation was identified in 18 of 118 isolates, all of which remained susceptible to echinocandin (File S1, sheet 1). These findings indicate that while mutations in the *FKS1* gene may be a primary cause of echinocandin resistance, not all isolates harboring these mutations demonstrate phenotypic resistance. The absence of these mutations in some resistant strains suggests that alternative mechanisms contribute to the altered phenotypes.

**TABLE 3:** Correlations of echinocandin resistance with hotspot *FKS1* mutations

Isolate ID	Source	*FKS1* mutation	Antifungal resistance MIC (µg/mL)			Cluster#
			Anidulafungin	Caspofungin	Micafungin	
CTO24-13	Skin swab	R1354H	0.5	0.25	0.25	7
CTO24-8	Urine	R1354S	>16	8	>8	6
CTO24-290	Urine	R1354S	4	2	4	13
CTO24-101	Urine	S639P	4	4	8	13
CTO24-427	Urine	S639P	4	4	4	1
CTO25-212	Wound	S639T	0.12	0.12	0.12	5
CTO23-77	Urine	S639Y	4	1	4	Unique
CTO23-210	Blood	M690I^*a*^	0.5	8	1	8
CTO23-240	Urine	M690I^*a*^	0.5	8	1	8
CTO25-208	Skin swab	M690I^*a*^	0.5	8	1	8
CTO25-249	Urine	M690I^*a*^	2	8	1	8
CTO25-254	Skin swab	None Detected	0.5	8	1	Unique

^*a*^ The *Candida auris* M690I mutation is not located in the primary hotspot regions (HS1 or HS2) of the *FKS1* gene. However, since it was the only mutation detected in these strains, it may contribute to the reduced susceptibility to caspofungin.

### Genetic variation among *C. auris* isolates within a single patient

During the study period, 12 patients had multiple *C. auris* isolates cultured, representing recurrent infections or persistent colonization. As shown in Table 4, the SNP distance between isolates from the same patient ranged from 0 to 14 over up to 744 days which is generally considered the same strain (18). In five patients, *C. auris* was initially detected in skin swabs and later recovered from peripheral blood and urine in as little as 15 days. Genomic analysis confirmed the same clone was present in both the initial skin swabs and subsequent infections, suggesting a progression from skin colonization to invasive disease. In another four patients, the same clonal strain identified in bloodstream infections was also found in the urinary and respiratory tracts, suggesting multisite colonization. Additionally, the same clone was carried by individual patients who had repeated urine and respiratory infections, suggesting persistent colonization and potential reservoir formation.

**TABLE 4:** Characteristics of repeated *Candida auris* infections in individual patients^*a*^

Case #	Age/sex /ethnicity	The maximum time between collections (days)	Number of isolates evaluated	Event	SNP variants	Designated cluster #	Outcome
				Colonization vs infection			
1	79/M/His	210	3	Skin swab—Respiratory—Bloodstream	0–3	1	In-hospital mortality
2	31/F/AA	16	2	Skin swab—Bloodstream	0–2	4	Clinically resolved
3	44/M/His	29	2	Bloodstream—Skin swab	0–3	4	In-hospital mortality
4	75/M/AA	54	2	Bloodstream—Respiratory	0–3	5	In-hospital mortality
5	44/M/His	744	3	Wound—Skin swab^*b*^	0–13	5	Clinically resolved
6	52/M/His	15	2	Skin swab—Bloodstream	0–1	6	In-hospital mortality
7	32/M/His	68	2	Skin swab—Urine	0–5	6	Clinically resolved
8	67/M/Whi	15	2	Bloodstream—Skin swab	0–3	6	Clinically resolved
9	72/M/His	13	2	Urine—Urine	0–0	7	In-hospital mortality
10	64/M/His	293	4	Respiratory—Skin swab—Respiratory—Bloodstream	0–14	7	In-hospital mortality
11	34/F/Whi	63	3	Skin swab—Bloodstream—Urine	0–2	8	In-hospital mortality
12	76/M/AA	144	2	Urine—Urine	0–9	13	Clinically resolved

^*a*^ Clinical and genomic characteristics of patients with repeated *Candida auris* isolations. The table summarizes patient demographics, specimen sources for sequential colonization or infection episodes, the maximum time between collections, number of isolates evaluated per patient, SNP variant ranges between paired isolates, assigned genomic cluster numbers and patient outcomes. The specimen types are listed in chronologic order of collection within each category, tracking the state progressed from colonization to infection. SNP, single-nucleotide polymorphism.

^*b*^ Two skin swab specimens were obtained at different times.

### Genetic variation among *C. auris* isolates within a single hospital

All 118 strains genotyped by WGS belonged to clade I of *C. auris*. To determine intra-clade variation and infer transmission patterns, a clonal analysis was conducted. The analysis revealed that 110 isolates belonged to 13 distinct clusters, while 8 isolates appeared to be unique strains. Using SNP matrix distance analysis (File S2), the 13 clusters were found to contain 2 to 18 isolates, representing 2 to 16 patients per cluster. The mean pairwise SNP distance was less than 34 within a cluster and 35–300 between clusters (18).

**Fig 1:**
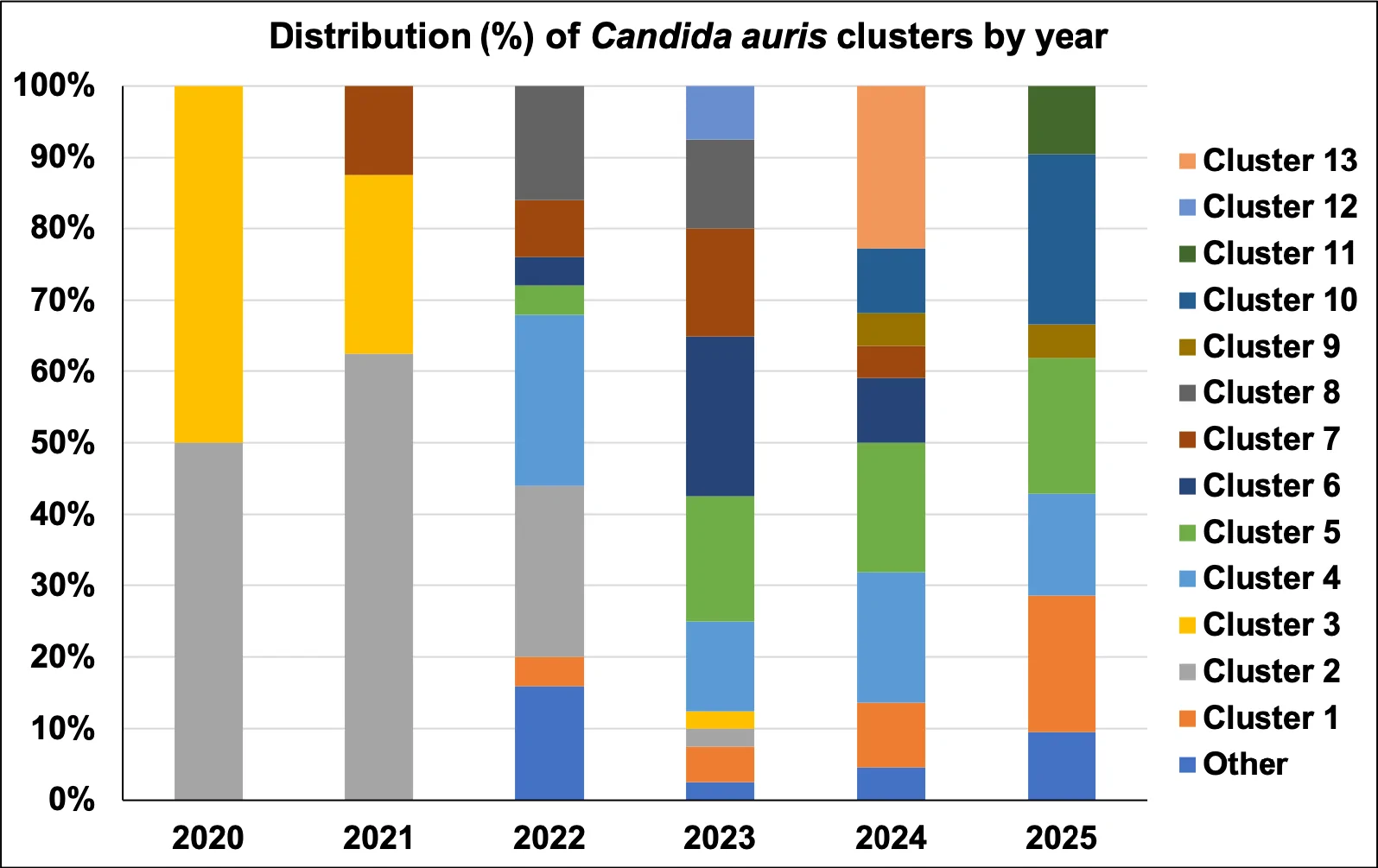
The percentage distribution of *C. auris* clusters in the hospital by year. A total of 118 isolates is included, with colored segments representing the percentage share of each cluster within a single year. Clusters 1 through 13 are distinguished by color, while isolates not belonging to these clusters are categorized as "other" to represent unique strains.

Fig. 1 illustrates the dynamic percentage distribution of *C. auris* clusters by year. Each year is represented as a single bar totaling 100%, divided into colored segments that show the percentage share of each cluster. Following the identification of two independent cases of *C. auris* infection in 2020, new external strains were introduced into the hospital yearly throughout the 6-year study period. Each year, multiple colored cluster groups appear with varying proportions within a single bar, indicating that multiple *C. auris* strains circulate concurrently. Many small, distinct clusters—such as clusters 11, 12, and 13—were introduced in 2025, 2023, and 2024, respectively. These represented time-limited, localized outbreaks that were resolved within a few weeks. Conversely, large clusters, such as clusters 1, 2, 4, 5, and 6, contain isolates from different patients and appear across multiple years. This suggests either a persistent hospital presence causing local patient-to-patient transmission or repeated introductions of the same strains. Detailed information on pairwise SNP distances among clusters is provided in Table S2

**Fig 2:**
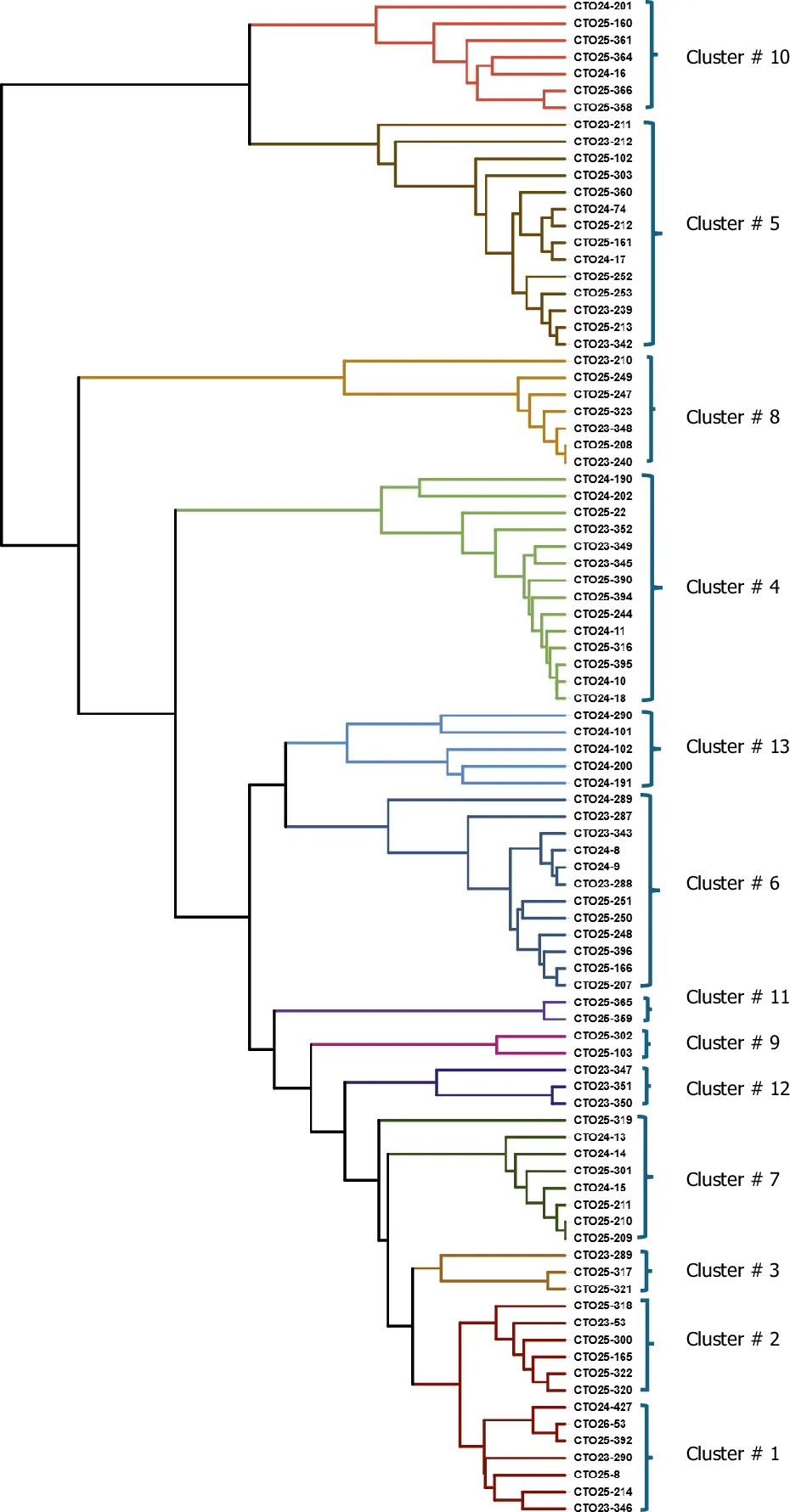
The existence of circulating clusters of *C. auris*. This maximum likelihood phylogenetic tree was generated based on the whole-genome sequencing of 90 clinical isolates collected between 2020 and 2025 at the 40× coverage threshold.

The phylogenetic relationships of these strains were analyzed on 90/110 isolates that met the 40× coverage threshold (shown in Fig. 2). The 13 clusters of genomes can be clearly separated, including three major clusters (clusters 4, 5, and 6) containing more than 10 isolates each, and smaller clusters containing at least 2 isolates. Finally, a high mortality rate of 64.3% (27/42) was observed across all clusters in patients with *C. auris* bloodstream infections.

## DISCUSSION

Clinical microbiology laboratories heavily rely on culture-based methods to identify *C. auris* and require mandatory antifungal susceptibility testing due to its high rates of resistance. Because no specific CLSI breakpoints exist, interpretation based on expert opinion and breakpoints established for closely related species can be challenging. Our data showed that 99% of clade I *C. auris* isolates are intrinsically resistant to fluconazole, while echinocandin resistance was seen in 7.6% and resistance to amphotericin B was identified in 54.2%. Previous studies have reported lower resistance to echinocandins, ranging from 1% to 4% (19, 20). Notably, most of our echinocandin-resistant isolates were recovered from urine samples, which is consistent with other findings (21). This higher resistance in urine is likely linked to the limited urinary excretion of echinocandins. While echinocandins are potent antifungal agents for bloodstream infections, they often do not reach significant therapeutic concentrations in the urine. This facilitates the development of resistance in this specific environment (21) and contributes to the fungus’s ability to spread in healthcare settings.

WGS provides an unambiguous method for characterizing *C. auris* isolates by detecting resistance mechanisms and enabling precise differentiation between isolates within a single *C. auris* clade. In this study, WGS provides additional support that resistance to fluconazole was linked to mutations in the *ERG11*, *CDR1,* and *TAC1B* genes, consistent with earlier reports (22, 23). *ERG11 and CDR1* mutations were identified in all isolates, while *TAC1B* mutations were present in 92.4% of isolates, highlighting their widespread role in azole resistance.

A recent report demonstrated that *ERG11* mutations correlate with fluconazole resistance and reduced fluconazole accumulation, a unique characteristic of *C. auris* (17). However, one of our isolates carried mutations in the *ERG11*, *CDR1*, and *TAC1B* genes but remained phenotypically susceptible to all azole drugs, indicating intraspecies variations and that identifying these mutations alone may not fully explain the observed phenotypes (24). Similarly, some isolates remain susceptible to voriconazole, isavuconazole, and itraconazole despite the presence of *ERG11*, *CDR1*, and *TAC1B* mutations. These susceptibility patterns are primarily due to (i) target site specificity, as specific mutations affect drug-binding affinity differentially; (ii) broader binding interactions, as different azoles interact with distinct chemical pockets and binding channels on the target enzyme (25). Consequently, these mutations do not automatically confer pan-azole cross-resistance. Furthermore, researchers have shown that fluconazole resistance can be driven by the overexpression of the ABC transporter gene *CDR1* due to activating mutations in the zinc cluster transcription factor *TAC1* (26). Additionally, the gene dosage of *PDR16*, which encodes a putative fungal phosphatidylinositol transfer protein, can directly modulate azole susceptibility in *C. auris* by altering drug response and reducing efficacy when overexpressed (27).

Our study also identified several novel mutations that appear to be cluster-related: *MRR1*-K381X, and *HMG1*-G765X in cluster 1 and 6; *MDR1*-I341V and *MRR1*-KD724N in cluster 4; *MRR1*-K493Ter in cluster 8; and a *FUR1*-G192R mutation in cluster 6. Although these variants have not yet been functionally characterized, the clustering of specific mutations within a *C. auris* clone suggests clonal microevolution (28).

Amphotericin B resistance in Candida species is typically caused by mutations in *ERG* genes, which lead to reduced plasma membrane ergosterol levels. In our study, 54% of the Clade I *C. auris* isolates showed MIC values of 2–3 µg/mL, which are above the CDC’s current clinical resistance threshold, yet no *ERG* gene mutations were identified. While a recent study reported that a deletion resulting in a frameshift in *ERG3* and a nonsense mutation in *ERG4* contribute to resistance in Clade III isolates, none of the isolates across Clades I–VI with MIC values at or slightly above the breakpoint contained an *ERG3* variant (29). This suggests that amphotericin B MIC values may vary based on the susceptibility testing method used and that epigenetic mechanisms may also contribute to resistance.

Although echinocandins are the recommended first-line treatment for invasive *C. auris* infections, resistance is rising due to acquired mutations in the *FKS1* gene, which alter the fungal cell wall’s target site. Our study confirmed that the isolates carrying the R1354S and S639P/Y mutations in the *FKS1* exhibited reduced susceptibility to anidulafungin, caspofungin, and micafungin. Conversely, strains carrying the previously described R1354H and S639T mutations (30, 31) remained susceptible to all three drugs. These susceptible results may indicate early-stage resistance development, a silent or non-hotspot variant, or heteroresistance, where resistant subpopulations are missed by standard testing (32).

Additionally, resistance to one echinocandin does not necessarily correlate with resistance to all (33). Our study found higher rates of resistance to caspofungin (nine isolates), compared to anidulafungin and micafungin (five isolates). This aligns with previous findings indicating that mutations altering specific residues impact resistance to certain echinocandins differently (34, 35). Interestingly, four of five isolates resistance to caspofungin alone carried M690I variant in the *FKS1* hotspot region 3 (10). This mutation was identified in a total of nine isolates clustered in Cluster 2 and Cluster 8, and these isolates showed up to three doubling dilution increases in modal MICs for micafungin compared to all other isolates. In contrast, all Cluster 4 isolates carrying the *FKS1*-V1818I mutation demonstrated no increase in micafungin MICs. The distinct role that each *FKS1* mutation plays in echinocandin resistance among these isolates remains to be elucidated.

While genotypic variation does not always predict observable traits due to environmental conditions, transcriptional regulation, and complex cellular mechanics, recent research highlights the role of the calcineurin signaling pathway in *C. auris* pathogenicity. Specifically, the deletion of the calcineurin complex impairs extreme thermotolerance and compromises cell membrane and wall integrity, increasing susceptibility to azoles and echinocandins (36). Additionally, reversible white-brown morphogenetic switching has been shown to drive phenotypic plasticity, altering stress tolerance and antifungal drug resistance (37).

Genomic analysis also confirmed clonal progression from colonization to invasive disease. In several cases, the same clonal strain identified on initial skin swabs was later detected in bloodstream infections just days later, suggesting a trajectory from colonization to systemic involvement. Additionally, patients experienced repeated urinary, respiratory, and bloodstream infections with the same clonal lineage initially identified, supporting persistent colonization and potential reservoir formation for recurring infections.

Previous studies have predominantly indicated that *C. auris* infections were hospital-acquired, with outbreaks often traced back to a single clonal lineage spreading within healthcare facilities (9). This understanding framed *C. auris* primarily as a nosocomial pathogen transmitted through contaminated hospital environments or direct patient contact. However, genomic analysis of isolates in our study revealed that genetically distinct *C. auris* strains can circulate concurrently within the same facility in a single year, driven by multiple independent introductions of different genotypes into the healthcare environment. This intra-clade genetic diversity suggests that many patients acquired the fungus from the community and were colonized prior to hospital admission. Furthermore, evidence points to subsequent patient-to-patient transmission occurring within healthcare settings, facilitating the spread of different clones among admitted individuals. These findings highlight the complex epidemiology of *C. auris* and emphasize the need for robust infection control measures both inside and outside hospital environments to prevent further dissemination.

While WGS is a powerful tool for high-resolution microbial identification, predicting antimicrobial resistance, and tracking hospital outbreaks, it has clear limitations. This is particularly true for intra-clade analysis in *C. auris*, where challenges such as low SNP resolution, structural and copy number variation masking, and reference genome bias can obscure transmission pathways and phenotype predictions (24).

### Conclusion

The presence of multiple *C. auris* strains with varying resistance patterns can pose challenges for clinicians in selecting appropriate antibiotics for treatment. Understanding the diversity of *C. auris* strains in a hospital setting is crucial for developing effective infection control measures, including pre-admission screening of high-risk patients, antimicrobial stewardship, and environmental cleaning protocols. Genomic analysis of *C. auris* isolates can help track the spread of multiple strains and efficiently identify outbreaks within hospitals.

## Data Availability

All read data have been deposited in the National Center for Biotechnology Information (NCBI) Sequence Read Archive (SRA) database under the Bioproject accession numbers PRJNA1140213 and PRJNA1426485.

## SUPPLEMENTAL MATERIAL

File S1 spectrum.01695-26-s0001.xlsx Mutations observed in clinical isolates.

File S2 spectrum.01695-26-s0002.xlsx SNP Distance Matrix table.

ASM does not own the copyrights to Supplemental Material that may be linked to, or accessed through, an article. The authors have granted ASM a non-exclusive, world-wide license to publish the Supplemental Material files. Please contact the corresponding author directly for reuse.
